# Editorial: Parkinson's disease: Technological trends for diagnosis and treatment improvement

**DOI:** 10.3389/fneur.2023.1151858

**Published:** 2023-02-14

**Authors:** Joan Cabestany, Antonio Suppa, Gearóid ÓLaighin

**Affiliations:** ^1^Department of Electronic Engineering, Universitat Politècnica de Catalunya (UPC), Barcelona, Spain; ^2^Sense4Care, Cornellà de Llobregat, Spain; ^3^Department of Human Neurosciences, Sapienza University of Rome, Rome, Italy; ^4^IRCCS Neuromed Institute, Pozzili, Italy; ^5^Department of Electric and Electronic Engineering, University of Galway, Galway, Ireland

**Keywords:** Parkinson's disease, inertial measurement unit, sensors, artificial intelligence, technology, image analysis, voice analysis

Parkinson's Disease (PD) is a very complex condition, presenting a wide range of motor and non-motor symptoms. It is considered the second neurodegenerative condition, after Alzheimer's Disease, in terms of prevalence, and some recent studies announce a drastic rising in the number of affected persons in the coming years. PD has no cure and its progression is always ineluctable. According to normal medical praxis, after the initial diagnosis, a pharmacological treatment is commonly started to improve the Quality of Life (QoL) of the affected person.

Nowadays, neurologists and related professionals can obtain benefits from the use of correctly addressed technologies. These possible benefits can help them in three different axes: (a) early detection of the disease for early initiation of the possible treatment, (b) obtaining additional objective information for a better adjustment of the treatment and (c) identification of candidates for advanced device-aided therapies (Second-line therapies—SLT) like Deep Brain Stimulation (DBS) or infusion pumps.

The present Research Topic entitled “*Parkinson's Disease: Technological Trends for Diagnosis and Treatment Improvement*” explores the contribution of technology, considered in a wide sense, to the improvement of healthcare services in the domain of PD. The 10 manuscripts included in this Research Topic deal with very different aspects and approaches, but all of them concern the improvement of the current treatments, the consideration of a new therapy or other considerations that can contribute to improving the QoL of the patients or their awareness about the disease.

A commonly reported and accepted difficulty that neurologists note, during the diagnosis and follow-up patients' visits, is the difficulty that many of them have to correctly identify and report their symptoms and the main states and temporal evolution of their condition. Timpka et al. present the conclusions about the observed discrepancies in the Home-diary annotations done by the patients participating in the study and those done by the qualified observers. The paper formulates a question about when the community will have the availability of an automatic or objective Home-diary, based on the technology. In this regard, the papers by Rodríguez*-*Martin et al., Zhang et al., and Geritz et al. contribute to this aspect. The first one presents a complete review of an already available CE-marked medical device that is commercialized to become a real Holter for the monitorization of the PD-related motor symptoms in real-life conditions. This solution is based on IMU and Machine Learning algorithms. In the other papers, the authors use IMU and MEMS-based technology for the identification of gait parameters permitting to distinguish and identify people affected by PD or to predict spatio-temporal walking parameters in hospitalized PD patients.

Apart from the above-mentioned technologies, many others can be applied and contribute to the early detection of PD and better diagnosis. For example, Suppa et al. address Machine Learning methods applied to voice disorders recognition for early assessment of PD and its monitoring along the treatment. An additional contribution is the proposal of a new score (the LR value) as a new measure of voice impairment. Another paper by Xu et al. applies phonation tests to persons affected by PD and makes a comparison with those done by healthy people. Comparison is done on the observation of the facial muscles' movement using a concrete commercial image treatment software platform. In another context, a better understanding of PD can be obtained when using signal processing analysis of the different bands in the EEG data of different PD patients. The paper contributed by Conti et al. extracts interesting conclusions on this topic, permitting the advance in the monitoring activity of recently diagnosed patients.

The rest of the papers contained in the Research Topic deals with complementary, but very promising usage of the technology to progress and improve the QoL of patients. For example, the Bianchini et al. paper explores the feasibility, safety and effectiveness of telerehabilitation in mild-to-moderate PD patients, demonstrating that remote physiotherapy programs could be viable and very useful to overcome situations with limited access to healthcare service (as a pandemic situation, for example).

Among SLT, DBS can be applied to selected advanced PD patients, but exhibits some problems in the process of programming and adjusting the stimulation parameters. Mei et al., apply image analysis techniques for the improvement of the DBS programming, concluding that using imaging-guided programming of directional DBS led to reducing programming time and the collection of side effects for the patients.

The last paper in the Research Topic, corresponds to Baek et al. on the possible future use of Focused Ultrasound (FUS) stimulation for the treatment of various brain diseases, including PD. The paper discusses future possible applications and the related challenges of this promising technology.

In conclusion, the contents of the present Research Topic allow us to be very confident with the existing and coming technologies. The main existing challenge is to completely overpass the still-existing adoption barriers. The benefits of the use of the technology will be evident when its adoption by all healthcare actors, including the patients, will be a reality.

## Author contributions

JC: conception, design, drafting, and critical revision of the editorial. AS: conception, design, and critical revision of the editorial. GÓ: critical revision of the editorial. All authors contributed to the article and approved the submitted version.

